# Summer and winter variations of BTEX concentrations in an oil refinery complex and health risk assessment based on Monte-Carlo simulations

**DOI:** 10.1038/s41598-023-37647-3

**Published:** 2023-07-01

**Authors:** Amir Hossein Khoshakhlagh, Saeid Yazdanirad, Mahdi Mousavi, Agnieszka Gruszecka-Kosowska, Mehran Shahriyari, Hassan Rajabi-Vardanjani

**Affiliations:** 1 Department of Occupational Health, School of Health, Kashan University of Medical Sciences, Kashan, Iran; 2 Social Determinants of Health Research Center, Shahrekord University of Medical Sciences, Shahrekord, Iran; 3 Department of Occupational Health, School of Health, Shahrekord University of Medical Sciences, Shahrekord, Iran; 4 Student Research Committee, Faculty of Health, Isfahan University of Medical Science, Isfahan, Iran; 5 Department of Environmental Protection, Faculty of Geology, Geophysics, and Environmental Protection, AGH University of Krakow, Al. Mickiewicza 30, 30-059 Krakow, Poland; 6 Unaffiliated, Tehran, Iran; 7 Department of Occupational Health, School of Health, Shahrekord University of Medical Sciences, Shahrekord, Iran

**Keywords:** Cancer, Health occupations

## Abstract

The summer and winter concentrations of BTEX pollutants were investigated in various workplaces of an oil Refinery, Iran. In total 252 air samples from the breathing zones of the following employees were collected: supervisors, safetymen, repairmen, site men, and all workers. Carcinogenic and non-carcinogenic risk values were calculated based on the USEPA methodology using Monte Carlo simulations. BTEX concentrations were higher in the summer than in the winter season for all workstations, especially for toluene and ethylbenzene. The mean values of exposure to benzene for repairmen and site men were higher than threshold limit value of 1.60 mg/m^3^ for both seasons. Non-carcinogenic risk (HQ) values calculated for summer season for benzene, ethylbenzene, and xylene in all workstations, as well as for toluene for repairmen and site men exceeded acceptable level of 1. In winter season the mean HQ values for benzene and xylene in all workstations, for toluene for repairmen and site men, and for ethylbenzene for supervisors, repairmen, and site men were also > 1. For all workstations definite carcinogenic risk was indicated as calculated LCR values for benzene and ethylbenzene exposure were higher than 1 × 10^–4^ in both summer and winter seasons.

## Introduction

The petroleum industry is considered worldwide as one of the most adversely affecting businesses the environmental and human health. This is due to the vast number of activities involved, like exploration and drilling, as well as due to the range of products being generated, namely oil, gas, and refinery and petrochemical products^1–3^. In oil-related industries various chemical compound are emitted^4^, among which volatile organic compounds (VOCs) are one of the most abundant^2^. Regarding that oil and petroleum products are the main sources of VOCs^5^, air pollution is the major concern related with industrial emissions. VOCs include a large group of hydrocarbons that can evaporate under atmospheric temperature and pressure due to high vapor pressure^6^ and might be emitted from the liquid phase into the air at room temperature^7^. Among VOCs, BTEX (benzene, toluene, ethylbenzene, and xylenes) compounds are of the most environmental concern. They are a prerequisite for the formation of photochemical oxidants^8^ and linked environmental effects, like formation of the tropospheric ozone, global warming, photochemical smog, and stratospheric ozone depletion^7^. Exposure to VOCs might be harmful to humans as they cause leukemia and adverse effects on the liver, nervous system, heart, and kidneys^9^. In addition, the International Agency for Research on Cancer (IARC) identified benzene to be the definite carcinogen to humans^5,10^. Due to the nature and working conditions leading to the employees’ exposure to BTEX, in the petroleum industry health safety is of particular importance^1^. As BTEX compounds are highly volatile VOCs, inhalation is the most important exposure pathway^6,11,12^. Chronic human exposure to benzene in workplaces might lead to bone marrow damage, which first manifests as anemia and leukopenia, and chronic contact with toluene might cause permanent damage to the central nervous system^2,13^. Ethylbenzene irritates the upper part of the respiratory system, might cause kidney damage and disorders of the cochlear part of the middle ear^14,15^. Xylene causes health disorders as the consequence of the upper respiratory tract, eyes, skin, and central nervous system irritation^14,15^.

The Oil Refinery complex, located in the southwestern Iran, currently supplies around 25% of the country’s fuel needs. The oil Refinery was built in 1912 by the Anglo-Persian Oil Company, later BP, as a pipeline terminus and was at the time one of the largest oil refineries^16^. After its destruction in 1980 during Iran–Iraq War and following rebuilding, the refinery’s production capacity increased steadily and with the current daily production of 15 million liters of gasoline is considered to be the largest oil refinery in Iran^16^, employing more than 8,000 workers in the three refining areas. The main products of the oil Refinery are liquefied petroleum gas, gasoline, kerosene, gas oil, jet fuel, fuel oil, base motor oil, bitumen, petroleum solvents, sulfur, naphtha, and associated gas^17^.

Accurate and timely identification and measurement of harmful chemical agents is very important in the prevention and in the early diagnosis of occupational diseases. Ultimately, it helps to maintain and improve employees’ health and to increase work efficiency^14,18^. Direct measurements of exposure determination to chemical substances in a personal respiratory area of working environments are the most reliable method^9^. By combining the data related to exposure and concentration of chemicals, the risk related to chemicals can be calculated^19^ and assessing the related health risks is the first step to adopt control measures for occupational exposure to these pollutants. Also, the emergence of new information about the adverse health effects of exposure to chemicals has caused risk assessment to be the powerful tool to quantify risk for regulatory purposes^20^.

Several studies investigated the occupational health risk related with the exposure to BTEX in various industries, in particular in petrochemical and oil refineries^2,3,5,21–24^, shipping port^25^, petrol station^26^, carpet manufacturing^14^, and municipal wastewater treatment plant^7^. Among these industries the highest carcinogenic and non-carcinogenic risk, described to be from unacceptable to very high, was reported for industries related with petrochemical processes. The studies described above revealed that the problem of BTEX emissions is observed in various countries and the health risk related with inhalational exposure to these pollutants among industrial workers poses unacceptable risk, both in terms of carcinogenic and non-carcinogenic effects.

Seasonal variations may change concentration patterns and peak time of pollutant occurrence^27^. Also, pollutant concentrations vary regarding the tasks performed by workers in the particular workstations^8^. Based on literature review we found out that majority of the available studies did not consider the impact of seasonal variations and concentration changes of investigated pollutants among various workstations during performing human health risk assessment. Thus, in the current study, specific attention was paid to investigate the effect of seasonal variations on the pollutant concentrations in the working environment, being the novelty of our studies. The main goal of the studies was to determine BTEX concentration changes regarding the different seasons and occupational workstations and assess the related human health risk due to inhalational exposure of employees in an Oil Refinery complex in Iran. The detailed objectives were to determine: (1) BTEX (benzene, toluene, ethylbenzene, and xylene) concentrations at following workstations: supervisors, safetymen, repairmen, site men, and all workers, (2) changes in BTEX concentrations during summer and winter periods, (3) BTEX concentration changes depending on the workstation type, and (4) health risk related with the inhalational exposure using Monte Carlo simulations.

## Materials and methods

### Sampling site description

In the oil refinery complex the BTEX compounds might be emitted from the following factory’s areas: catalytic reforming unit, kerosene transportation area, new transportation area, gasoline post treatment unit, alkylation unit, wastewater treatment plant, and distillation units. In these units four types of workers are employed, namely site men, repair men, safetymen, and supervisors. Due to wear and tear of oil storage, transmission, and refining systems as well as due to the age of this complex, the exposure of workers to BTEX can be a result of petroleum leakages from tanks and pipes.

### Occupational exposure characteristic

The cross-sectional study was performed on the employees of the distillation units of an Oil Refinery, Iran. The inclusion criteria for the refinery’s workers to participate in the study was to have at least 12 months of work experience and to be exposed to BTEX compounds via inhalational pathway based on preliminary examination. The number of subjects required in this study was computed using Cochran equation with error level of 0.05 and was equal to 84. From each workstation three air samples were taken for the each participant in order to compute the time weighted average (TWA) during the shiftwork exposure, thus in total 252 samples were collected. To check the impact of the used methodology and equipment on pollutant concentration measured, also 25 blank samples were taken in the same conditions as the main samples. Prior the investigations participants signed the informed consent. Questionnaire surveys performed included answering the questions on age, weight, work experience, number of working days per year, and exposure duration per day.

### Air sampling and exposure assessment

#### Sampling method

Concentrations of BTEX compounds, were measured using the National Institute for Occupational Safety and Health (NIOSH) 1501 method^28^. The measurements were conducted during winter and summer of the year 2021^29^. To collect the air samples in the working environments, the adsorbent tube containing activated coconut charcoal (front: 100 mg and rear: 50 mg) produced by SKC Inc. were used^30^. The adsorbent tubes were placed on the workers’ collars within the breathing zone, and a calibrated SKC pump with a flow rate from 50 to 200 ml/min was applied to pass air through the tube^30^. Sampling was carried out by a personal sampling pump, model AirChek TOUCH (5–5000 mL/min, SKC, Inc.). Initially, a pretest was performed to reveal the breakthrough volume. For each subject three indoor air samples were gathered to cover the work shift, thus measurements were performed at the beginning, in the middle, and at the end of the work shift of the participants included^31^. The sampling time of each sample was between 80 and 120 min. After sampling, the plastic caps were used to seal adsorbent tubes. The samples were transferred to a laboratory in a cool box to prevent the potential leakage. The atmospheric conditions of the sampling locations were measured using WBGT device (Tenmars electronics CO., Taiwan) and the results of air temperature and relative humidity measurements were recorded.

#### Sample preparation and analysis

In this step, the adsorbent tubes were broken and the pollutants were chemically desorbed by 1 ml of carbon disulfide solvent in extraction vials. After 60 min, 1 μl of this solution was injected by a syringe into a gas chromatograph with a capillary column equipped with a GC-FID flame ionization detector (GC 7890 Agilent)^30^. Helium (flow rate of 1 ml/min) was exploited as carried gas. Moreover, 25 blank samples were analyzed to eliminate errors during sampling and analyzing steps.

#### Quality assurance/quality control (QA/QC)

Both the air and blank samples were placed in a cold box with ice packs (~ 4 °C) after sampling and during the transfer to the laboratory. After reaching the laboratory, samples were stored in the temperature of 4 °C before the analyses. The sampling tubes were broken at the sampling site, then their ends were sealed using plastic caps, and transmission and analyses were performed using the similar methods in the case of both main and blank samples. Extraction solvent of carbon disulfide (CS_2_) was injected and analyzed using the gas chromatography flame ionization detector (GC-FID) three times to determine BTEX contents. The limit of detection (LOD) was determined as follows: LOD = 3.3 × standard deviation (SD) of the blanks (slope of the calibration curve)^32^. Moreover, a pre-set concentrations of BTEX were prepared and entered the adsorbent (charcoal) tubes. The mean recovery percentage for BTEX compounds was equal to 92 ± 14%.

### Health risk assessment

Quantitative risk assessment method developed by the United States Environmental Protection Agency (USEPA) is used to assess the risk related with the exposure to chemical substances. Depending on the health effects, carcinogenic or non-carcinogenic, Lifetime Cancer Risk (LCR) index or Hazard Quotient (HQ) were calculated in our study, respectively.

#### Non-carcinogenic risk assessment

To evaluate the non-carcinogenic risk of BTEX compounds, the United States Environmental Protection Agency (USEPA) method was used^33^. Non-carcinogenic risk is described by the Hazard Quotient (HQ) values, that are defined as the ratio between exposure to investigated pollutant and the reference value, expressing the maximum daily exposure that should not cause negative health effects. In the inhalational exposure pathway the Eq. (1) was used:

1 $${\text{HQ }} = {\text{ EC}}/{\text{RfC}},$$

where EC is exposure concentration of the investigated pollutant (mg/m^3^) and RfC is reference concentration (mg/m^3^).

In order to calculate the EC value the Eq. (2)^34^ was used:

2 $${\text{EC}} = \left( {{\text{C}} \times {\text{ET}} \times {\text{ED}} \times {\text{EF}}} \right){\text{/AT}},$$

where C is the concentration of pollutant (mg/m^3^), ET is exposure time (hours/day), ED is exposure duration (years), EF is exposure frequency (days/year), AT is averaging time (ED in years × 365 days/year). The values of exposure and toxicological parameters used in the study are presented in Table 2.

If HQ values are < 1 it indicates the lack of adverse non-carcinogenic health effects, while HQ values ≥ 1 point the presence of non-carcinogenic health effects^35^.

#### Carcinogenic risk assessment

For determination of the carcinogenic risk of pollutants also the USEPA method was used. In this method the carcinogenic risk is described by the lifetime cancer risk (LCR) index. As no Inhalational Unit Risk (IUR) values were available for toluene, ethylbenzene, and xylene, instead of exposure concentration (EC), chronic daily intake (CDI) was used in order to receive the unified results. The LCR values were calculated according to the Eq. (3):

3 $${\text{LCR }} = {\text{ CDI }} \times {\text{ SF}},$$

where CDI is chronic daily intake (mg/kg-day) and SF is cancer slope factor ((mg/kg-day)^–1^) . Values of SF was taken from the IRIS toxicological database^36,37^, however only SF values for benzene and ethylbenzene were available. The CDI values were computed using the Eq. (4)^34^.

4 $$\mathrm{CDI }= \frac{\mathrm{C }\times \mathrm{IR }\times \mathrm{ED }\times \mathrm{EF }}{\mathrm{BW }\times \mathrm{AT }},$$

where C is concentration of pollutant (mg/m^3^), IR is inhalation rate (m^3^/day), ED is exposure duration (years), EF is exposure frequency (days/year), BW is body weight (kg), AT is averaging time (days). The values of exposure and toxicological parameters used in the study are presented in Table 1.

**Table 1:** Exposure and toxicological parameters used in this study for non-carcinogenic and carcinogenic risk assessment.

Parameter	Description	Value	Reference
IR (m^3^/day)	Inhalation rate, adult	16	EPA 2011
ET (hours/day)	Exposure time	8–12	Questionnaire
EF (days/year)	Exposure frequency	234–270	Questionnaire
ED (years)	Exposure duration	21–40	Questionnaire
BW (kg)	Body weight	70–97	Questionnaire
AT (ED in years × 365 days/year in days)	Averaging time	9000	USEPA 2009
RfC (mg/m^3^)	Reference concentration from inhalation	Benzene: 0.03, toluene: 5.00, ethylbenzene: 1.00, xylene: 0.10	IRIS database
SF (mg/kg-day)^–1^	Slope factor	Benzene: 0.029, ethylbenzene: 0.0087	IRIS database

Calculated LCR values higher than 1 × 10^–4^ indicate definite risk, LCR between 1 × 10^–4^ and 1 × 10^–5^ indicate probable risk, LCR values between 1 × 10^–5^ and 1 × 10^–6^ indicate possible risk, and LCR values lower than 1 × 10^–6^ indicate negligible risk^2^.

### Monte-Carlo simulations

To achieve as reliable and realistic results as possible regarding the uncertainty and variability of the measurements performed, mathematical modeling is recommended^38^. Monte Carlo simulation (MCS), as a probabilistic and statistical-mathematical approach, that uses a combination of simulations, in order to determine uncertainty by the Monte Carlo method. The computations were conducted by 1000 iterations, and the results were estimated with a confidence degree between 1 and 99%^39^. In this study, Crystal Ball software (version 11.1.2.4, Oracle, Inc., USA) was exploited for Monte Carlo simulations (MCS).

### Statistical analysis

In order to check the normality of the data distribution, the Kolmogorov–Smirnov test was used. Given the lack of normal distribution of the variables, Friedman test was used to examine difference of mean values of exposure to BTEX in two investigated seasons, namely summer and winter. Other values of descriptive statistics were reported using SPSS software.

### Ethics statement

The Research Ethics Committee of Shahrekord University of Medical Sciences (SKUMS) granted ethics approval for this study (IR.SKUMS.REC.1401.021). Also, we confirm that all methods were performed in accordance with the relevant guidelines and regulations.

### Consent to participate

All of the subjects had full consent to participate in the current study.

## Results and discussion

### Atmospheric conditions of the performed measurements

In our studies measurements of air temperature, relative humidity, and wind speed were performed along with the BTEX concentrations. In the summer season the mean value of air temperature was equal to 40.4 °C (standard deviation, SD 1.89 °C). In the winter season the mean air temperature was reported to be 15.3 °C (SD 1.64 °C). The mean value of the relative humidity in the summer season was equal to 45.1% with SD of 5.11%. In the winter season the mean value of the relative humidity was equal to 20.5% (SD 2.12%). The measurements of wind speed in summer season indicated the mean value equal to 1.88 m/s (SD 1.04 m/s). In the winter season the mean value of wind speed was reported to be 1.96 m/s, with the SD value of 0.89 m/s. To sum up, the measurements revealed that temperature and relative humidity at workplaces were much higher in summer than in winter seasons, while wind speed was higher in winter than in summer season.

### Seasonal variations of BTEX concentrations

**Figure 1:**
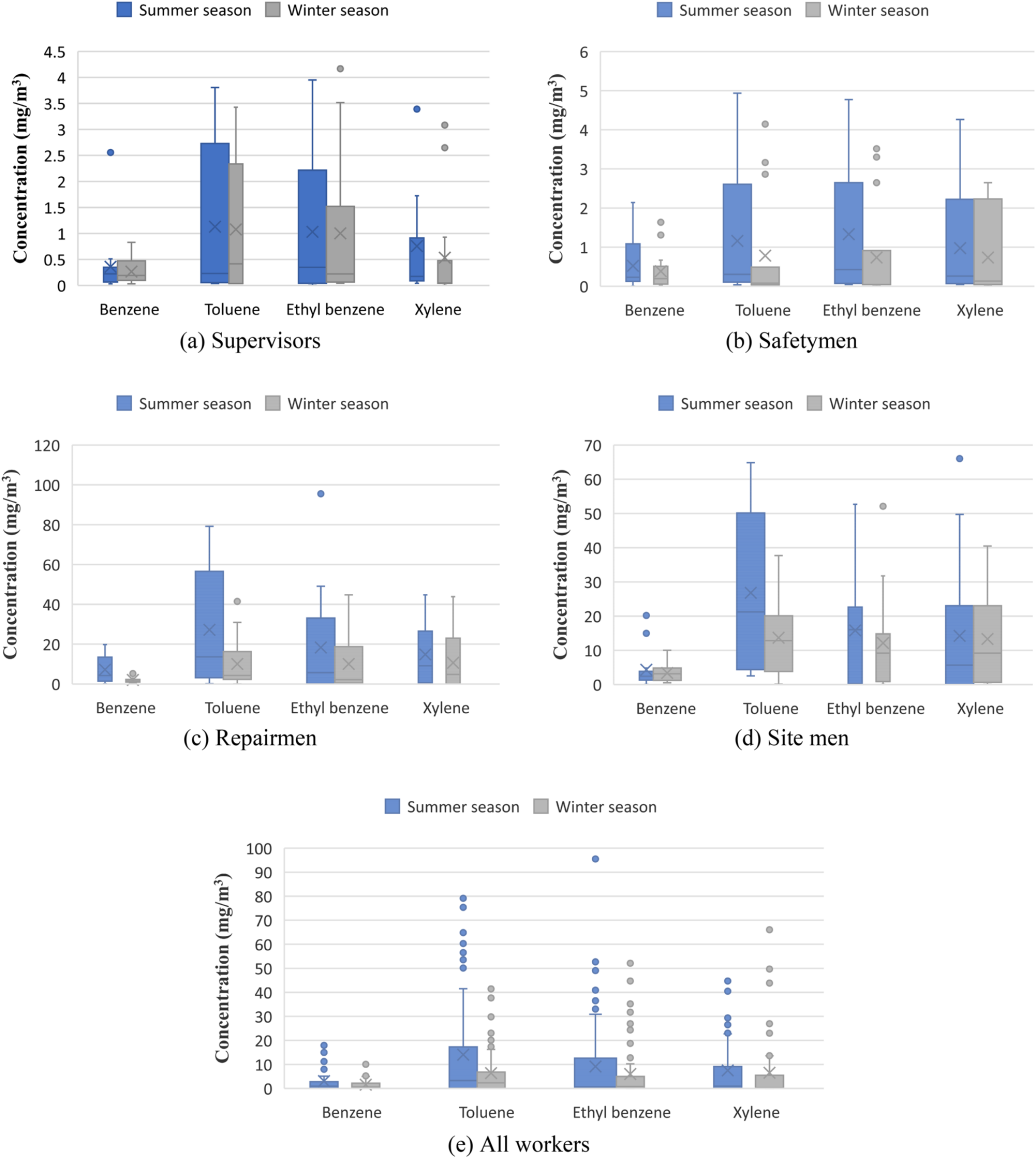
Concentration of BTEX (mg/m^3^) in the breathing zones of the various workstations in summer and winter seasons.

The concentrations of BTEX in the breathing zones of the employees in the various workstations in summer and winter seasons are presented in Fig. 1. It can be observed that analyzed BTEX concentrations were higher in the summer than in the winter season for all workstations and were particularly visible for toluene and ethylbenzene. It can be related with the fact that the mean temperature in the investigated production process was 40.4 °C in summer and 25.3 °C in winter affecting the higher vaporization of the BTEX compounds. Also, the relative humidity in summer season, which mean value was equal to 45.1%, while in winter season it was equal to 20.5% also might affected the increased BTEX emissions. The results of Friedman test revealed that exposure to xylene in supervisors and exposure to BTEX in safetymen, repairmen, and site men were significantly higher in summer season compered to winter seasons (P < 0.045) (Table 2). Moreover, it was revealed that the exposure in particular workstations was ordered decreasingly as follows: repairmen > site men > safety men > supervisors. In the particular groups of employees, the mean BTEX values in summer and winter seasons in the breathing zones were as follows (mean summer vs mean winter concentration, mg/m^3^): repairmen: benzene 7.193 vs 2.023, toluene 27.200 vs 10.070, ethylbenzene 18.326 vs 10.027, xylene 14.833 vs 10.615; site men: benzene 4.311 vs 3.369, toluene 26.791 vs 13.685, ethylbenzene 15.822 vs 12.135, xylene 14.242 vs 13.325; safetymen: benzene 0.522 vs 0.384, toluene 1.160 vs 0.782, ethylbenzene 1.333 vs 0.734, xylene 0.975 vs 0.735; supervisors: benzene 0.359 vs 0.267, toluene 1.129 vs 1.078, ethylbenzene 1.032 vs 1.002, xylene 0.755 vs 0.533. For all working groups the mean BTEX values in summer and winter seasons in the breathing zones were as follows (mean summer vs mean winter concentration, mg/m^3^): benzene 3.073 vs 1.534, toluene 14.070 vs 6.404, ethylbenzene 9.121 vs 5.982, and xylene 7.472 vs 6.531.

**Table 2:** Statistical description to BTEX exposure (mg/m^3^) in various workstations in summer and winter seasons.

Work-station	Pollutant	Summer season			Winter season			P value
		Range	Mean	Standard deviation	Range	Mean	Standard deviation	
		mg/m^3^						
Supervisors	Benzene	0.032–2.556	0.359	0.626	0.032–0.831	0.267	0.228	0.052
	Toluene	0.038–3.806	1.129	1.508	0.038–3.429	1.078	1.294	0.197
	Ethylbenzene	0.004–3.951	1.032	1.342	0.043–4.168	1.002	1.441	0.071
	Xylene	0.043–3.391	0.755	1.042	0.004–3.083	0.533	0.986	0.002
Safetymen	Benzene	0.003–2.140	0.522	0.660	0.032–1.636	0.384	0.485	0.039
	Toluene	0.038–4.937	1.160	1.705	0.038–4.145	0.782	1.380	0.001
	Ethylbenzene	0.043–4.776	1.333	1.639	0.004–3.517	0.734	1.285	0.002
	Xylene	0.043–4.264	0.975	1.379	0.004–2.649	0.735	1.078	0.008
Repairmen	Benzene	0.073–19.775	7.193	7.166	0.351–7.539	2.023	1.944	0.001
	Toluene	0.136–79.139	27.200	29.304	0.038–41.454	10.070	12.467	0.001
	Ethylbenzene	0.043–95.522	18.326	26.886	0.043–44.722	10.027	14.537	0.033
	Xylene	0.043–44.722	14.833	15.257	0.004–43.853	10.615	13.323	0.008
Site men	Benzene	0.064–20.190	4.311	5.622	0.511–10.031	3.369	2.540	0.043
	Toluene	2.540–64.856	26.791	22.749	0.113–37.685	13.685	11.958	0.001
	Ethylbenzene	0.043–52.668	15.822	15.783	0.043–52.103	12.135	14.422	0.031
	Xylene	0.043–66.041	14.242	19.846	0.043–40.467	13.325	13.172	0.045
All workers	Benzene	0.003–20.190	3.073	5.307	0.032–10.031	1.534	2.045	0.001
	Toluene	0.037–79.139	14.070	22.310	0.038–41.454	6.404	10.188	< 0.001
	Ethylbenzene	0.043–95.522	9.121	17.231	0.004–52.103	5.982	11.284	< 0.001
	Xylene	0.043–44.722	7.472	11.908	0.004–66.041	6.531	13.158	< 0.001

Investigations of Hawari et al.^40^ performed in urban areas of Malaysia indicated positive correlations of BTEX concentrations with relative humidity, however negative correlations with wind speed, solar radiation, and air temperature. Study of Popitanu et al.^41^ in the ambient air of Arad city, Romania, revealed that the increase of the BTEX concentrations in winter season was related with the usage of fuels in the central heating season. Also, due to the higher reaction rates with OH radicals in the atmosphere BTEX were removed faster in summer than in winter^41^. The studies of Bodor et al.^4^ on seasonal differences in air pollution around Ploiesti oil refining complex in Romania also pointed that the highest.

BTEX values were measured in cold season, particularly in winter. Investigations of Seco et al.^42^ in the forest site in the Western Mediterranean Basin revealed that almost all analyzed VOCs had higher average mixing ratios during the summer than the winter season, except for VOCs that are linked to anthropogenic sources: for them lower (benzene) or similar (toluene) mixing ratios in summer than in winter were stated.

For all the workstations the decreasing order of BTEX was as follows: toluene > ethylbenzene > xylene > benzene. These finding stay in line with investigations of Rajabi et al.^43^ indicating that among all VOCs emitted from crude oil, toluene, benzene, hexane, heptane, cyclohexane, and pentane were found to be highly detected and concentrated compounds. Investigations of Ercan et al.^44^ in Istanbul, Turkey revealed that BTEX concentrations in industrial zones were higher than those measured in suburban locations. Investigations of Bretón et al.^45^ on BTEX concentrations in an urban site located in the vicinity of an oil storage-distribution facility in Paraiso, Tabasco, Mexico revealed that lower BTEX concentrations in the dry season during midday and morning were consistent with increased photochemical activity during these hours. Moreover, higher ambient temperatures coupled with calm periods and low rainfall during the dry season could cause higher BTEX emissions. These studies also revealed that BTEX concentrations during dry season would be expected to be reduced by photochemical degradation, as this season is characterized by high solar radiation intensity^45^. Finally, the low wind speeds during dry season provided unfavorable dispersion conditions resulting in higher BTEX concentrations^45^. Jiang et al.^46^ in their studies ordered the seasonal variation of the mean BTEX concentrations as follows: winter > spring > autumn > summer, pointing that besides sources' strength, the seasonal and diurnal variations of atmospheric BTEX in urban areas were also strongly dependent on meteorological conditions and photochemical activity.

As most of the studies presented above discussed the BTEX concentration changes in the ambient air in the vicinity of industrial sources and traffic emission it cannot be compared directly to our studies. In our research BTEX concentrations in the breathing zones were investigated, where the only source of emission was the presence of the refinery productions processes. Studies of Tabari et al.^25^ in Mahshahr oil shipping port, Khuzestan province, Iran revealed that the total emission of VOCs were equal to 933.25 tons/year with the main emission sources to be storage tanks, pump houses, and wastewater treatment pool. BTEX emissions were equal to 1.49 tons/year of benzene, 3.2 tons/year of toluene, 0.57 tons/year of ethylbenzene, and 1.53 tons/year of xylenes^25^. Thus, seasonal variability of the BTEX concentrations in our studies were primarily related with the emissions in the refinery and local atmospheric conditions in the petrochemical processing.

### BTEX concentrations and threshold limit values

**Figure 2:**
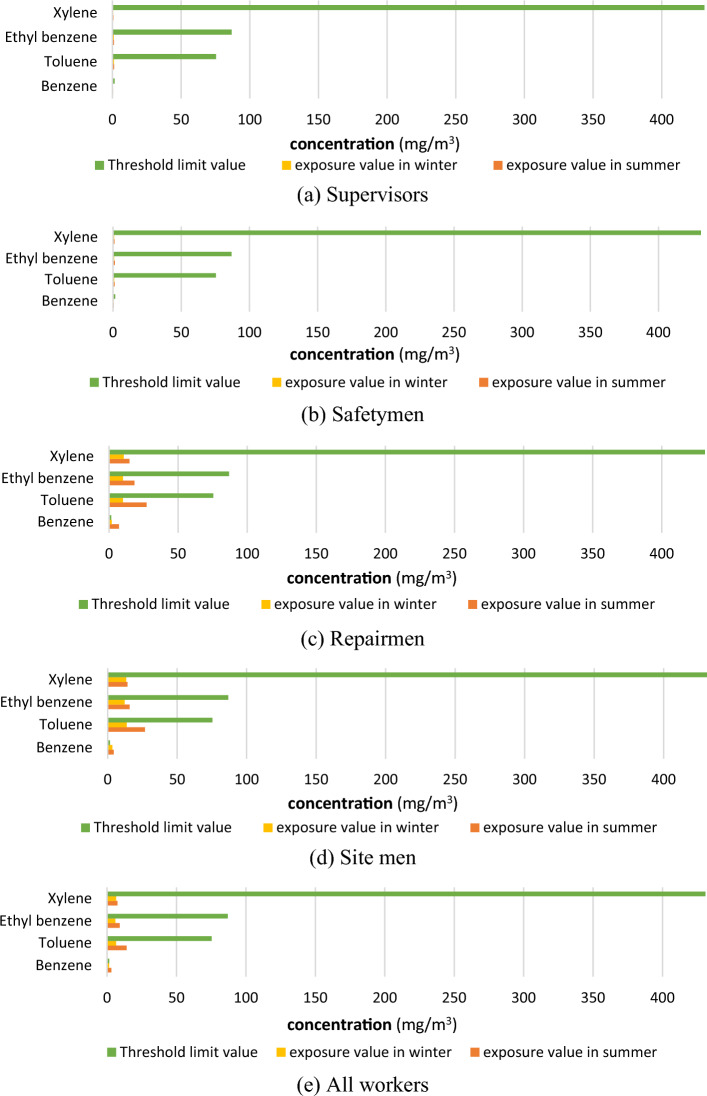
Comparison of mean BTEX exposure values and threshold limit values (TLVs).

Mean exposure values of BTEX were compared with the threshold limit values (TLVs) in various workstations in summer and winter seasons (Fig. 2). The American Conference of Governmental Industrial Hygienists (ACGIH) recommends the following threshold limit values (TLVs) for exposure to BTEX compounds: benzene 60 mg/m^3^ (0.5 ppm), toluene 175.37 mg/m^3^ (20 ppm), ethylbenzene 86.84 mg/m^3^ (20 ppm), and xylene 434.19 mg/m^3^ (100 ppm)^47^. As the threshold limit values (TLVs) are recommended for 8 h of work per day and for 5 days of work per week, the Scala brief model was used for correcting the amount of threshold limit value – time-weighted average (TLV-TWA) if work duration was longer than 40 h per week^47^. The results of our investigations revealed that the mean values of exposure to benzene for repairmen and site men workstations were higher than recommended TLV equal to 1.60 mg/m^3^ for both summer and winter seasons. Considering all workers, the mean value of exposure to benzene was higher than TLV equal to 1.60 mg/m^3^ only for the summer season.

Our results stay in line with the findings of Moradpour et al.^8^, who investigated 40 VOCs in the Mahshar Petroleum industrial zone, Khuzestan province, Iran located about 100 km east from the investigated in our studies. In these studies, it was revealed that benzene concentrations in Mahshar Petroleum facility exceeded the threshold limit value for occupational exposure^8^. Our outcomes stay also in line with the investigations of Jalilian et al.^48^, who demonstrated in the study on BTEX effect on blood and spirometry parameters among staff in the Oil Refining Company in Iran, that toluene, ethylbenzene, and xylenes, concentrations in the breathing zones were lower than the TLV-TWA recommended by ACGIH, while the average concentration of benzene exceeded the allowable limit. Thus, it is recommended that facilities like petrochemical plants were equipped in extensive air monitoring to ensure that exposure is below occupational limits, especially for workplaces with higher levels of benzene, where the frequency of controls should be increased^49^. Various means of protective measures against exposure to BTEX compounds, like adequate engineering, management controls, and periodic inspection, are suggested to protect the employee’s health^48^.

### Non-carcinogenic risk assessment

**Figure 3:**
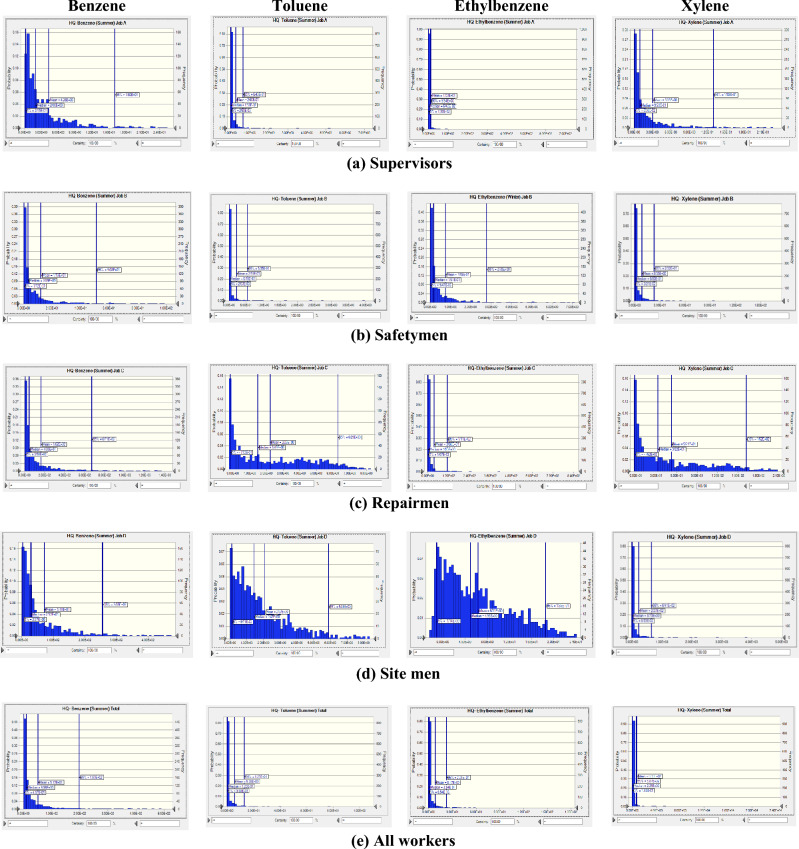
Probability distribution and percentile related to non-carcinogenic risk of exposure to BTEX in summer season.

**Figure 4:**
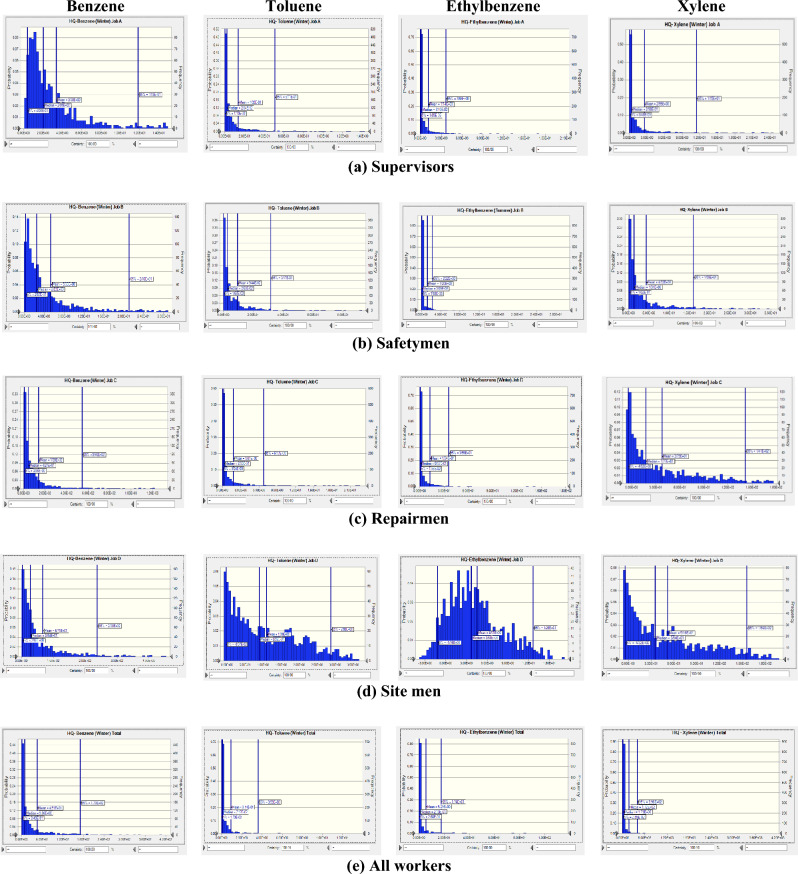
Probability distribution and percentile related to non-carcinogenic risk of exposure to BTEX in winter season.

The probability distributions and percentiles related to non-carcinogenic risk (HQ) of exposure to BTEX for various workstations in summer (Fig. 3) and in winter (Fig. 4) seasons were calculated using Monte Carlo simulation. In Table 3 the statistical description of HQ values for BTEX in various workstations in summer and in winter seasons as a heat map was presented. Our results for summer season revealed that the mean values of calculated HQ for benzene in all workstations (> 4.26), for toluene for repairmen and site men (> 2.07), for ethylbenzene in all workstations (> 1.20), and for xylene in all workstations (> 3.03) were above acceptable level of 1. In winter season also, the mean values of computed HQ for benzene in all workstations (> 3.38), for toluene for repairmen and site men (> 1.09), for ethylbenzene for supervisors, repairmen, and site men (> 1.14), and for xylene in all workstations (> 2.55) were above 1. Regarding all workers, only the mean value of calculated HQ for toluene in winter season (0.914) was below the acceptable level of 1.

**Table 3:**
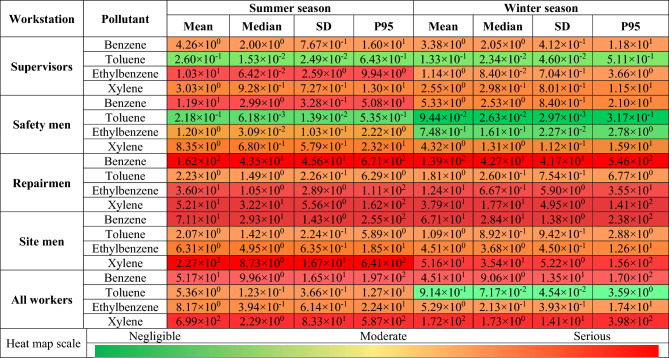
Non-carcinogenic risk (HQ) values for BTEX exposure in various workstations in summer and winter seasons as a heat map, P95–95th percentile.

Results revealed that highest mean value of HQ for supervisors was related to exposure to ethylbenzene (10.3) in summer season and benzene (3.38) in winter season. For safetymen, the highest mean value belonged to benzene exposure in summer season (11.90) and in winter season (5.33). For repairmen also, the highest mean value was related to exposure to benzene in summer season (162.00) and winter season (139.00). For site men, the highest mean HQ value was assigned to xylene exposure in summer season (227.00) and benzene in winter season (67.10). For all workers, the highest mean HQ values were related to xylene exposure in summer season (699.00) and in winter season (172.00). In all workstations, the mean HQ value in summer season was higher than in winter season.

In summer season, the highest mean values of estimated HQ for benzene (162.00), toluene (2.23), and ethylbenzene (36.00) were related to exposure of repairmen, while the highest mean value of estimated HQ for xylene (227.00) belonged to exposure of site men. In winter season also, the highest mean values of estimated HQ were stated for benzene (139.00), toluene (1.81), and ethylbenzene (12.4), while the highest mean value of estimated HQ for xylene (51.60) belonged to exposure of site men.

Considering HQ values in occupational exposure to BTEX in refinery industry it was revealed that all calculated values indicated moderate to serious non-carcinogenic risk. Only for supervisors and safetymen exposure to toluene, for all workers in winter season, and for ethylbenzene exposure for safety men in winter season the risk was negligible.

### Carcinogenic risk assessment

**Figure 5:**
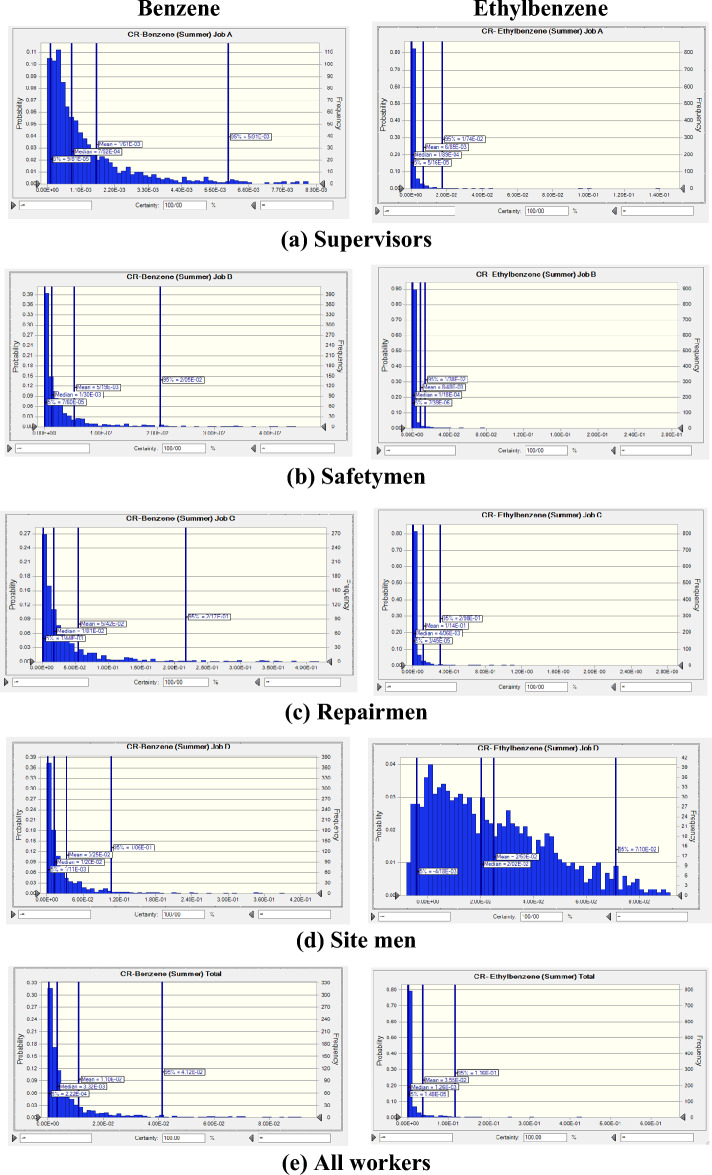
Probability distribution and percentile related to carcinogenic risk of exposure to benzene and ethylbenzene in summer season.

**Figure 6:**
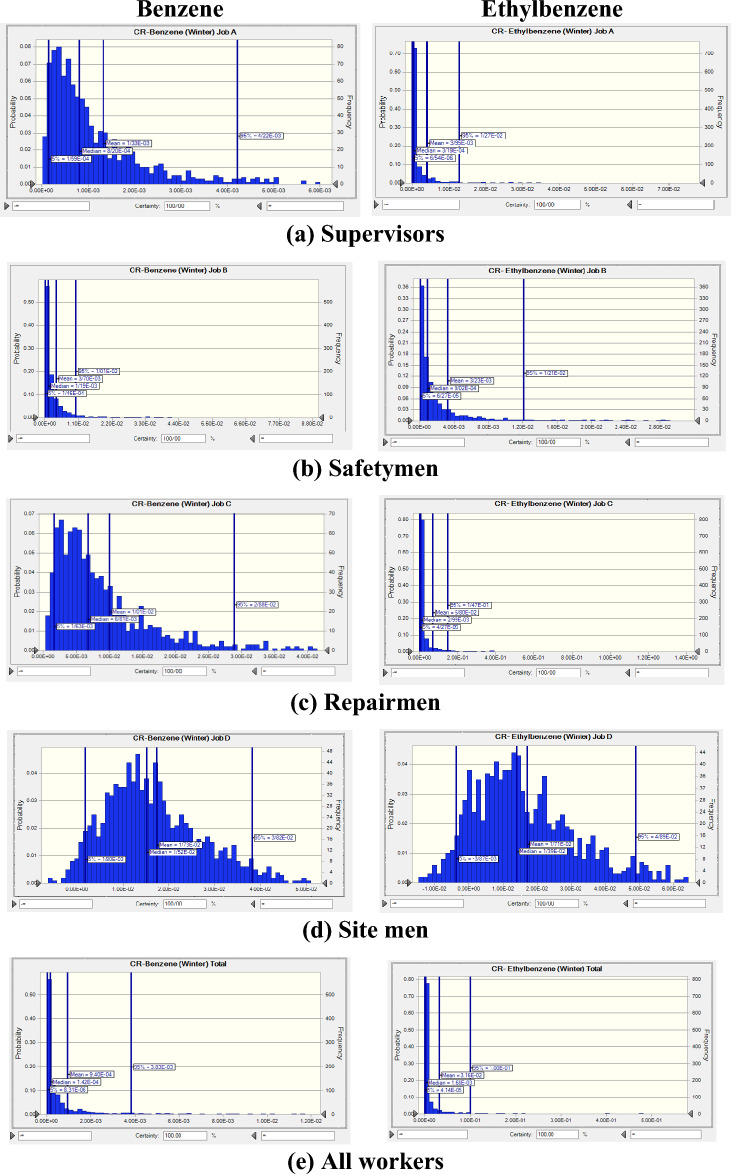
Probability distribution and percentile related to carcinogenic risk of exposure to benzene and ethylbenzene in winter season.

The probability distributions and percentiles related to carcinogenic risk (LCR) of exposure to BTEX for various workstations in summer (Fig. 5) and in winter (Fig. 6) seasons were also performed using Monte Carlo simulation. In Table 4 the statistical description of LCR values for BTEX in various workstations in summer and in winter seasons as a heat map was presented. The results showed that the mean LCR values for benzene and ethylbenzene were higher than 1 × 10^–4^ for all workstations in both summer and winter seasons. It indicated that there was a definite carcinogenic risk for workers exposed to benzene and ethylbenzene in all workstations of the investigated company in both seasons. Due to the lack of SF values for toluene and xylene from toxicological databases, only calculations for benzene and ethylbenzene were presented.

**Table 4:**
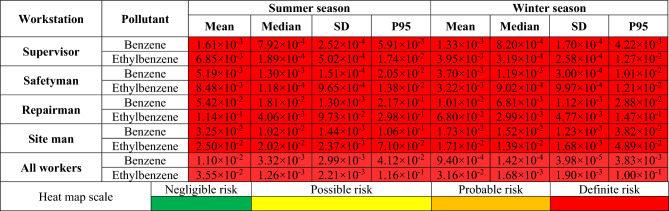
Carcinogenic risk (LCR) values for benzene and ethylbenzene in various workstations in summer and winter seasons as a heat map, P95–95th percentile.

Results indicated that the highest mean LCR value for supervisors was related to ethylbenzene exposure in summer (6.85 × 10^–3^) and in winter (3.95 × 10^–3^) seasons. For safetymen, the highest mean LCR value belonged to ethylbenzene exposure in summer season (8.48 × 10^–3^) and to benzene exposure (3.70 × 10^–3^) in winter season. For repairmen also, the highest mean value was related to benzene exposure in summer season (5.42 × 10^–2^) and ethylbenzene in winter season (6.80 × 10^–2^). For site men, the highest mean value was assigned to benzene exposure in summer (3.25 × 10^–2^) and in winter (1.73 × 10^–2^) season. For all workers, the highest mean LCR values were related to ethylbenzene in summer (3.55 × 10^–2^) and in winter (3.16 × 10^–2^) season. Considering all workstations, the mean LCR value in summer season was higher than in winter season.

In summer season, the highest mean values of estimated LCR for benzene (5.42 × 10^–2^) and ethylbenzene (1.14 × 10^–1^) were related to workstation of repairmen. In winter season also, the highest mean value of estimated LCR for benzene (1.73 × 10^–2^) was related to site men, while the highest mean value of estimated LCR for ethylbenzene (6.80 × 10^–2^) belonged to repairmen.

Considering all LCR values in occupational exposure to BTEX in refinery industry it was revealed that all calculated values indicated definite carcinogenic risk, namely for all workstation, for benzene and ethylbenzene compounds, and for both summer and winter seasons.

### Risk assessment in other studies

Results of our research stay in line with several other studies on human health risk related with VOCs and BTEX occupational exposure in petrochemical industry.

The semi-quantitative risk assessment performed by Hoseini et al. in the an Iranian oil refinery revealed very high risk levels of benzene for workers in the pit area, high risk levels for toluene, and moderate risk levels for xylene and paraxylene. Regarding the carcinogenic risk assessment benzene was stated the main compound among VOCs responsible for significant health risk^5^. Studies of Mihajlović et al.^49^ on occupational exposure to BTEX in petrochemical plant in Serbia revealed that on average the employees were at high carcinogenic risk with a potential risk value equal to 6.10 × 10^–3^, with special concern to workers exposed to benzene concentrations higher than 18 mg/m^3^.

Research of Tong et al.^23^ on health risk related with exposure to VOCs in a petrochemical refinery in Hainan, China revealed that benzene, toluene, ethyl benzene, and xylene were the primary pollutants. For non-carcinogenic risk the highest HQ values were related with the aromatic hydrocarbon extraction device (AHED). Carcinogenic risk values exceeded the acceptable in the case of benzene exposure, indicating BTEX posing the health risk to workers.

Results of risk assessment performed by Heibati et al.^3^ in major oil distribution company in Iran revealed that the mean carcinogenic risk related with exposure to benzene were 16.08 for tanker loading workers, 2.47 for tank-gauging workers, 0.20 for drivers, 0.21 for firefighters, and 0.06 for office workers. Non-carcinogenic risk was revealed to be related to benzene and xylene exposure^3^. Results of Zhang et al.^2^ performed in the petroleum refinery in the Pearl River Delta, China indicated the total non-carcinogenic risk in the basic chemical area the highest (3.1 × 10^3^), owing to the highest level of total concentration of VOCs. The carcinogenic risk was reveled definite as the total carcinogenic risk ranged from 2.93 × 10^–3^ in the wastewater treatment area to 1.1 × 10^–2^ in the basic chemical area^2^. Investigations of Kitwattanavong et al.^26^ analyzing the occupational exposure of petrol stations workers in the Inner City of Bangkok, Thailand revealed the high carcinogenic risk due to inhalational exposure as the risk levels for benzene were in the range 1.82 × 10^–4^–2.50 × 10^–4^, and for ethylbenzene: 4.11 × 10^–6^–5.52 × 10^–6^, exceeding the acceptable risk level.

On the contrary, some research did not confirm our findings. These were mainly related with the health risk analysis in the vicinity of the BTEX emission facilities, indicating that their concentrations were dispersed and thus not posed the significant health risk for inhabitants of these regions. Ercan et al.^44^ indicated that the health risk assessment in the Istanbul city for benzene was equal to 7.71 × 10^–7^ and non-carcinogenic risk for toluene, ethylbenzene, and xylenes was lower than the acceptable level of 1. In addition, health risk analysis performed among workers of the municipal wastewater treatment plant in Shiraz, Iran^7^ indicated that carcinogenic risk values for all sampling sites were below the threshold limit of 1.0 × 10^–4^. Regarding the non-carcinogenic risk only for benzene the risk was exceeding the acceptable level of 1, while for toluene the risk values were lower than 1. The results of Zhang et al.^22^ performed in Lanzhou, the major industrial areas of China revealed that non-carcinogenic risk related with airborne benzene exposure had acceptable HQ values for adults (0.185) and for children (0.102). However, a high carcinogenic risk (> 10^−4^) from benzene in several sampling sites was revealed that diffuse distance become significant for carcinogenic risk.

## Conclusions

Investigation on seasonal variations of BTEX concentrations in an Oil Refinery in Iran indicated that for all workstations investigated VOCs concentrations were higher in the summer than in the winter season. The decreasing order of BTEX concentrations in workstations was as follows: toluene > ethylbenzene > xylene > benzene. Benzene concentrations in breathing zones exceeded the threshold limit value TLV for repairmen and site men workstations for both summer and winter seasons. For all workers, the mean value of exposure to benzene was higher than TLV only in the summer season. Occupational inhalational exposure to all BTEX compounds revealed moderate to serious non-carcinogenic risk for all workstations in summer season. Carcinogenic risk from exposure to benzene and ethylbenzene in all workstations was stated as definite in both summer and winter seasons. Based on the results of our studies it is recommended to apply effective protection measures among workers of the oil refinery industry and to interest the responsible authorities and managers of this industry to introduce systemic solutions to protect employees’ health.

## Data Availability

The datasets used and/or analyzed during the current study are available from the corresponding author upon reasonable request.

## References

[1] Gardner R (2003). Overview and characteristics of some occupational exposures and health risks on offshore oil and gas installations. Ann. Occup. Hyg..

[2] Zhang Z, Yan X, Gao F, Thai P, Wang H, Chen D (2018). Emission and health risk assessment of volatile organic compounds in various processes of a petroleum refinery in the Pearl River Delta, China. Environ. Pollut..

[3] Heibati B, Pollitt KJG, Karimi A, Charati JY, Ducatman A, Shokrzadeh M (2017). BTEX exposure assessment and quantitative risk assessment among petroleum product distributors. Ecotoxicol. Environ. Saf..

[4] Bodor K, Szép R, Bodor Z (2022). Time series analysis of the air pollution around Ploiesti oil refining complex, one of the most polluted regions in Romania. Sci. Rep..

[5] Hoseini LK, Yengejeh RJ, Rouzbehani MM, Sabzalipour S (2022). Health risk assessment of volatile organic compounds (VOCs) in a refinery in the Southwest of Iran using SQRA method. Front. Public Health.

[6] Wang H, Nie L, Li J, Wang Y, Wang G, Wang J (2013). Characterization and assessment of volatile organic compounds (VOCs) emissions from typical industries. Chin. Sci. Bull..

[7] Dehghani M, Mohammadpour A, Abbasi A, Rostami I, Gharehchahi E, Derakhshan Z (2022). Health risks of inhalation exposure to BTEX in a municipal wastewater treatment plant in Middle East city: Shiraz, Iran. Environ. Res..

[8] Moradpour Z, Shahna FG, Bahrami A, Soltanian A, Hesam G (2017). Evaluation of volatile organic compounds at petrochemical complexes in Iran. Health Scope.

[9] Yu B, Yuan Z, Yu Z, Xue-song F (2022). BTEX in the environment: An update on sources, fate, distribution, pretreatment, analysis, and removal techniques. Chem. Eng. J..

[10] Song S-K, Shon Z-H, Kang Y-H, Kim K-H, Han S-B, Kang M (2019). Source apportionment of VOCs and their impact on air quality and health in the megacity of Seoul. Environ. Pollut..

[11] Abd Hamid HH, Jumah NS, Latif MT, Kannan N (2017). BTEXs in indoor and outdoor air samples: Source apportionment and health risk assessment of benzene. J. Environ. Sci. Public Health.

[12] Montero-Montoya R, López-Vargas R, Arellano-Aguilar O (2018). Volatile organic compounds in air: Sources, distribution, exposure and associated illnesses in children. Ann. Glob. Health.

[13] Farris GM, Robinson SN, Gaido KW, Wong BA, Wong VA, Hahn WP (1997). Benzene-induced hematotoxicity and bone marrow compensation in B6C3F1 mice. Fundam. Appl. Toxicol..

[14] Khoshakhlagh AH, Morais S (2022). Volatile organic compounds in carpet manufacturing plants: Exposure levels and probabilistic risk assessment using Monte-Carlo simulations. Hum. Ecol. Risk Assess. Int. J..

[15] Davidson CJ, Hannigan JH, Bowen SE (2021). Effects of inhaled combined benzene, toluene, ethylbenzene, and xylenes (BTEX): Toward an environmental exposure model. Environ. Toxicol. Pharmacol..

[16] Wikipedia. National Iranian oil refining and distribution company. https://en.wikipedia.org/wiki/National_Iranian_Oil_Refining_and_Distribution_Company (2023).

[17] International I. Blast Rocks Iran’s Oldest Oil Refinery Near Persian Gulf. https://www.iranintl.com/en/202209022189 (2022).

[18] Beheshti M, Firoozi Chahak A, Alinaghi Langari A, Rostami S (2015). Semi-quantitative risk assessment of health exposure to hazardous chemical agents in a petrochemical plant. J. Occup. Health Epidemiol..

[19] Nieuwenhuijsen M, Paustenbach D, Duarte-Davidson R (2006). New developments in exposure assessment: the impact on the practice of health risk assessment and epidemiological studies. Environ. Int..

[20] Guo H, Lee S, Chan L, Li W (2004). Risk assessment of exposure to volatile organic compounds in different indoor environments. Environ. Res..

[21] Jalilian S, Sabzalipour S, Mohammadi Rouzbahani M, Rajabzadeh Ghatrami E, Ibrahimy GL (2022). Health risk assessment of occupational exposure of refinery unit site workers to BTEX in an oil refinery company. J. Health Sci. Surveill. Syst..

[22] Zhang T, Kang W, Ge X, Lin Q, Chen Q, Yu Y (2022). Explication on distribution patterns of volatile organic compounds in petro-chemistry and oil refineries of China using a species-transport model and health risk assessment. Sci. Total Environ..

[23] Tong R, Yang Y, Shao G, Zhang Y, Dou S, Jiang W (2019). Emission sources and probabilistic health risk of volatile organic compounds emitted from production areas in a petrochemical refinery in Hainan, China. Hum. Ecol. Risk Assess. Int. J..

[24] Chang M-Y, Hsu Y-S, Chen H-S (2021). Choice experiment method for sustainable tourism in Theme Parks. Sustainability.

[25] Raazi Tabari M, Sabzalipour S, Peyghambarzadeh S, Jalilzadeh R (2020). Vapor loss of volatile organic compounds (VOCs) from the shipping port of abadan petroleum refinery. Pollution.

[26] Kitwattanavong M, Prueksasit T, Morknoy D, Tunsaringkarn T, Siriwong W (2013). Health risk assessment of petrol station workers in the inner city of Bangkok, Thailand, to the exposure to BTEX and carbonyl compounds by inhalation. Hum. Ecol. Risk Assess. Int. J..

[27] Pandey J, Agrawal M, Khanam N, Narayan D, Rao D (1992). Air pollutant concentrations in Varanasi, India. Atmos. Environ. B Urban Atmos..

[28] Schlecht PC, O'Connor P (2003). NIOSH Manual of Analytical Methods.

[29] Tulashie SK, Addai EK, Annan J-S (2016). Exposure assessment, a preventive process in managing workplace safety and health, challenges in Ghana. Saf. Sci..

[30] Eller P (1994). NIOSH Manual of Analytical Methods) Hydrocarbons.

[31] Leidel NA (1977). Occupational Exposure Sampling Strategy Manual.

[32] Desimoni E, Brunetti B (2015). About estimating the limit of detection by the signal to noise approach. Pharm. Anal. Acta.

[33] Agency USEP. Risk assessment guidance for superfund Volume I: Human health evaluation manual (Part F, supplemental guidance for inhalation risk assessment). In OoSRaT (ed) Innovation, DC2009.

[34] Tong R, Ma X, Zhang Y, Shao G, Shi M (2018). Source analysis and health risk-assessment of ambient volatile organic compounds in automobile manufacturing processes. Hum. Ecol. Risk Assess. Int. J..

[35] Sadeghi-Yarandi M, Karimi A, Ahmadi V, Sajedian AA, Soltanzadeh A, Golbabaei F (2020). Cancer and non-cancer health risk assessment of occupational exposure to 1, 3-butadiene in a petrochemical plant in Iran. Toxicol. Ind. Health.

[36] Vallero DA (2016). Environmental Biotechnology.

[37] Risk assessment guidance for superfund (RAGS): Part F (2019).

[38] Agency USEP. Use of Monte Carlo Simulation in Risk Assessments Region 3 Technical Guidance Manual, Risk Assessment (2022).

[39] Badeenezhad A, Radfard M, Passalari H, Parseh I, Abbasi F, Rostami S (2019). Factors affecting the nitrate concentration and its health risk assessment in drinking groundwater by application of Monte Carlo simulation and geographic information system. Hum. Ecol. Risk Assess. Int. J..

[40] Hawari NSSL, Latif MT, Abd Hamid HH, Leng TH, Othman M, Mohtar AAA (2022). The concentration of BTEX in selected urban areas of Malaysia during the COVID-19 pandemic lockdown. Urban Clim..

[41] Popitanu C, Cioca G, Copolovici L, Iosif D, Munteanu F-D, Copolovici D (2021). The seasonality impact of the BTEX pollution on the atmosphere of Arad City, Romania. Int. J. Environ. Res. Public Health.

[42] Seco R, Peñuelas J, Filella I, Llusià J, Molowny-Horas R, Schallhart S (2011). Contrasting winter and summer VOC mixing ratios at a forest site in the Western Mediterranean Basin: The effect of local biogenic emissions. Atmos. Chem. Phys..

[43] Rajabi H, Mosleh MH, Mandal P, Lea-Langton A, Sedighi M (2020). Emissions of volatile organic compounds from crude oil processing–Global emission inventory and environmental release. Sci. Total Environ..

[44] Ercan Ö, Dinçer F, Ceylan Ö (2019). Spatial and seasonal variations of atmospheric BTEX, sulfur dioxide, nitrogen dioxide, and ozone concentrations in Istanbul, and health risk assessment of BTEX. Environ. Forensics.

[45] Bretón RMC, Bretón JGC, Kahl JW, Chi MPU, Lozada SEC, de la Luz Espinosa Fuentes M (2022). Seasonal and diurnal variations of BTEX in Ambient air from a site impacted by the oil industry in Southeast Mexico. Bull. Environ. Contam. Toxicol..

[46] Jiang Z, Grosselin B, Daële V, Mellouki A, Mu Y (2017). Seasonal and diurnal variations of BTEX compounds in the semi-urban environment of Orleans, France. Sci. Total Environ..

[47] Values ATL, editor and Biological Exposure Indices (BEIs) 2012. 354 The American Conference of Governmental Industrial Hygienists (ACGIH) (2021).

[48] Jalilian S, Sabzalipour S, Rouzbahani MM, Ghatrami ER, Ghavamabadi LI (2022). Assessing the effect of BTEX on blood and spirometry parameters staff in a petroleum refinery. Front. Public Health.

[49] Mihajlović V, Grba N, Suđi J, Eichert D, Krajinović S, Gavrilov MB (2021). Assessment of occupational exposure to BTEX in a petrochemical plant via urinary biomarkers. Sustainability.

